# Expression of calcium-binding protein S100A2 in breast lesions

**DOI:** 10.1054/bjoc.2000.1488

**Published:** 2000-11-22

**Authors:** D Liu, P S Rudland, D R Sibson, A Platt-Higgins, R Barraclough

**Affiliations:** School of Biological Sciences, University of Liverpool, P.O. Box 147, Liverpool, L69 7ZB, UK; Clatterbridge Cancer Research Trust, J.K. Douglas Laboratories, Clatterbridge Hospital, Bebington, Wirral, CH63 4JY, UK

**Keywords:** S100A2, breast cancer, in situ hybridization, carcinoma in situ, suppression subtractive hybridization

## Abstract

A suppression subtraction cDNA library representing mRNAs expressed at a higher level in a benign breast tumour-derived cell line relative to the malignant MCF-7A cell line contained cDNAs corresponding to mRNAs for plasminogen activator inhibitor I, annexin VIII and the EF-hand protein S100A2. S100A2 protein has previously been shown to be expressed in normal human breast epithelium, but not in human breast carcinoma cell lines. Using a PCR-based assay and in situ hybridization on histological sections of human breast specimens, the mRNA for S100A2 was shown to be present in all benign breast lesions examined as well as in normal epithelium. S100A2 mRNA was detectable in 37% of specimens of carcinoma in situ, but in less than 15% of carcinoma specimens. The results suggest that the loss of S100A2 is associated with the development of malignant cells and is not associated with early tumour development. © 2000 Cancer Research Campaign http://www.bjcancer.com


*British Journal of Cancer* (2000) **83**(11), 1473–1479
© 2000 Cancer Research Campaign
doi: 10.1054/ bjoc.2000.1488, available online at http://www.idealibrary.com on IDEAL®

### Expression of calcium-binding protein S100A2 in breast lesions

**D Liu[1], PS Rudland[1], DR Sibson[2], A Platt-Higgins[1] and R Barraclough[1]**

[1]School of Biological Sciences, University of Liverpool, P.O. Box 147, Liverpool, L69 7ZB, UK; [2]Clatterbridge Cancer Research Trust, J.K. Douglas Laboratories, Clatterbridge Hospital, Bebington, Wirral, CH63 4JY, UK

Summary A suppression subtraction cDNA library representing mRNAs expressed at a higher level in a benign breast tumour-derived cell line relative to the malignant MCF-7A cell line contained cDNAs corresponding to mRNAs for plasminogen activator inhibitor I, annexin VIII and the EF-hand protein S100A2. S100A2 protein has previously been shown to be expressed in normal human breast epithelium, but not in human breast carcinoma cell lines. Using a PCR-based assay and in situ hybridization on histological sections of human breast specimens, the mRNA for S100A2 was shown to be present in all benign breast lesions examined as well as in normal epithelium. S100A2 mRNA was detectable in 37% of specimens of carcinoma in situ, but in less than 15% of carcinoma specimens. The results suggest that the loss of S100A2 is associated with the development of malignant cells and is not associated with early tumour development. © 2000 Cancer Research Campaign http://www.bjcancer.com

**Keywords:** S100A2; breast cancer; in situ hybridization; carcinoma in situ; suppression subtractive hybridization

The S100 family of proteins consists of at least 13 EF-hand containing proteins which exhibit a cell type-specific distribution in normal cells (Schäfer and Heizmann, 1996). Five of these proteins, S100A2–S100A6 are clustered within 18 kbp of one another on chromosome 1 of the human genome (Pedrocchi et al, 1994). Although the functions of these S100 proteins are not known with any certainty (Donato, 1999), for some, their altered expression has been associated with cancer cells. For example, high S100A6 levels in melanomas is associated with decreased survival of patients (Maelandsmo et al, 1997). Elevated levels of S100A4 are strongly associated with the ability of tumour cells to metastasize in cell and transgenic mouse models of breast cancer (Davies et al, 1993, 1996; Ambartsumian et al, 1996), and furthermore, immunocytochemically detected S100A4 in the tumours from an archival group of 349 breast cancer patients has recently been shown to be associated with early patient death (Platt-Higgins et al, 2000; Rudland et al, 2000). However, in contrast to the up-regulation of these two S100 proteins in cancer cells, the mRNA for S100A2 has been reported to be abundant in cells cultured from normal human breast, but to be absent in breast carcinoma-derived cells (Lee et al, 1992). The loss of expression of S100A2 in cancer cell lines relative to normal breast-derived cells has been linked to increased methylation of the S100A2 gene in the cancer cells (Lee et al, 1992; Wicki et al, 1997). However, a tumour biopsy sample subjected to genomic sequencing suggested that not all cells contained methylated DNA in the promoter region of the S100A2 gene, probably reflecting heterogeneity of the specimen (Wicki et al, 1997). Using transactivation assays, it has been shown that the S100A2 promoter can be transcriptionally activated by wild-type, but not by mutant p53 (Tan et al, 1999).

As part of a programme of identifying genes that are differentially expressed between human benign and malignant breast cell lines using suppression subtractive hybridization, we recently identified S100A2 mRNA as one that was present at a higher level in the benign relative to the malignant cells, suggesting that S100A2 is produced in benign tumour cells. Thus, using in situ hybridization, the distribution of S100A2 mRNA has been sought in benign breast lesions, carcinomas in situ and carcinoma specimens. The results have been related to the p53 status of the specimens.

### MATERIALS AND METHODS

### Human breast tissue and cell lines

Human breast specimens were obtained from the CANDIS Cancer Tissue Bank Research Centre and the Royal Liverpool University Hospital (Liverpool, UK) as described previously (Anandappa et al, 1994; Taylor et al, 1997). Characteristics of the specimens are shown in Table 1. Immunocytochemical detection of p53 was carried out as described previously (Platt-Higgins et al, 2000).

The normal human mammary epithelial cell line, Huma 7, subcloned from primary cultures derived from reduction mammoplasty specimens of normal breast tissue which had been immortalized with SV40 virus (Rudland et al, 1989), the benign human mammary epithelial cell line, Huma 123, and the derivative benign human mammary myoepithelial-like cell line, Huma 109 (Ke et al, 1993), derived from HMT-3522 which was itself derived from a primary cell culture of human fibrocystic disease displaying prominent epithelial hyperplasia (Briand et al, 1987), the malignant human mammary epithelial cell lines derived from pleural effusions of breast cancer patients, MCF-7A, T-47D, ZR-75 and MDA-MB-231 (Engel and Young, 1978) were cultured as described previously (Rudland et al, 1989; Ke et al, 1993).

*Received 2 May 2000*
*Revised 31 July 2000*
*Accepted 14 August 2000*

*Correspondence to:* R Barraclough

**Table 1** Characteristics of tumour specimens

| | |
|---|---|
| Total number of normal specimens | 8 |
| Of which from normal breast | 3 |
| Of which from specimens with no residual carcinoma | 5 |
| Total number of benign specimens[a] | 15 |
| Of which p53 positive | 2 |
| Of which p53 negative | 11 |
| Of which of unknown p53 status | 2 |
| Total number of carcinoma specimens | 27 |
| Of which invasive ductal carcinoma and DCIS | 19 |
| Of which p53 positive | 12 |
| Of which p53 negative | 11 |
| Of which of unknown p53 status | 4 |

[a]Benign specimens consisted of 7 fibroadenomas and 8 fibrocystic diseases, which included hyperplasias of usual type.

### Subtractive hybridization

A suppression subtracted (Diatchenko et al, 1996) library consisting of polymerase chain reaction (PCR) products representing mRNAs expressed at a higher level in the benign human breast-derived cell line, Huma 123, relative to the malignant breast epithelial cell line MCF-7A was constructed using a PCR-Select™ cDNA Subtraction Kit (Clontech, Palo Alto, California, USA), according to the manufacturer's methodology.

A blunt-ended vector was prepared from pBluescript $KS^-$ (Stratagene, La Jolla, USA) which was digested to completion with *Eco*RV (Roche Molecular Biochemicals, Mannheim, Germany). The digested plasmid DNA was purified by phenol/chloroform extraction and ethanol precipitation (Sambrook et al, 1989). The linear, blunt-ended plasmid DNA was tailed with 10 μM dideoxythymidine triphosphate (ddTTP) (Pharmacia, Uppsala, Sweden) using 100 units of terminal transferase (Roche Molecular Biochemicals, Mannheim, Germany) in the buffer supplied by the manufacturer at 37˚C for 1.5 h. The ddT-tailed vector was purified by phenol/chloroform extraction, followed by ethanol precipitation.

PCR products constituting the subtracted library were ligated into the ddT-tailed pBluescript vector using T4 DNA ligase (New England Biolabs, Hertfordshire, UK) in the buffer supplied by the manufacturer and incubated at 5–8˚C overnight. *E. coli* cells XL-1 Blue (Stratagene, La Jolla, USA) were transformed by electroporation (2.5 kV, 25 μFD, 200 Ω, time constant 4–5) using a Gene Pulser unit (Bio-Rad laboratories, Hertfordshire, UK), and plated onto agar plates containing 100 μg $ml^{-1}$ ampicillin, and 40 μM isopropyl-β-D-thiogalactopyranoside (IPTG), 32 μg $ml^{-1}$ 5-bromo-4-chloro-3-indolyl-β-D-galactoside (X-gal) for blue/white colour selection. Randomly picked individual positive colonies from the selection plates were grown overnight in LB medium containing 100 μg $ml^{-1}$ ampicillin in 96-well plates at 37˚C and their plasmid DNA analysed by reverse Northern blotting to confirm the differential expression of the picked clones.

### Reverse Northern screening of the subtracted cDNA libraries

One μl of each culture was amplified by PCR in 20 μl reactions using the Advantage KlenTaq Polymerase Mix (Clontech, Palo Alto, California, USA). More than 98% of the randomly picked clones contained inserts. After amplification, PCR products were subjected to agarose gel electrophoresis and the amount of DNA determined from the intensity of the ethidium bromide-stained bands. Equal amounts of PCR products were combined with a measured amount of bromophenol blue dye solution, denatured and blotted onto duplicate Nylon membranes (Amersham, Bucks, UK) using a 48-well slot-blot apparatus (Anachem-Scotlab, Luton, UK). Filters were checked that each slot contained the same intensity of dye. The filters were hybridized (Sambrook et al, 1989) with equivalent amounts of double-stranded cDNA derived from driver (MCF-7A) and tester (Huma 123) mRNA respectively which had been labelled to approximately equal specific activity ($1 \times 10^9$ d.p.m.$\mu g^{-1}$ DNA) with [$\alpha^{32}$P] dCTP (ICN, Hampshire, UK). After being washed twice for 15 min each at room temperature with 2 × SSPE (1 × SSPE = 180 mM NaCl, 10 mM sodium phosphate, 1 mM EDTA, pH 7.4), 0.1% (w/v) sodium dodecyl sulphate (SDS), twice for 15 min each at 68˚C with 1 × SSPE, 0.1% (w/v) SDS, and twice for 20 min each at 68˚C with 0.1 × SSPE, 0.1% (w/v) SDS, filters were exposed to X-ray film (Kodak X-AR 5) with an intensifying screen at −80˚C overnight. The resultant autoradiographic images were analysed using IMAGE software (NIH, Bethesda, USA) on a Macintosh computer.

### Northern blotting

Total cellular RNA was prepared using the guanidinium-isothiocyanate-caesium chloride method (Chirgwin et al, 1979; Barraclough et al, 1987; Han et al, 1987). Poly(A)-containing RNA was isolated from total RNA using the Fast Track mRNA isolation kit (Invitrogen, Groningen, Netherlands). Gel electrophoresis of 10 μg aliquots of total RNA in formaldehyde and Northern blotting was performed as described previously (Sambrook et al, 1989), and filters were washed as for reverse Northern hybridization. The S100A2 cDNA probe was the same as the 258 bp probe used for in situ hybridization (see below) and was labelled by random-primed DNA synthesis (Feinberg and Vogelstein, 1984) using a labelling kit (Roche Molecular Biochemicals, Mannheim, Germany) to $1 \times 10^9$ d.p.m.$\mu g^{-1}$ DNA. The constitutive probe, 36B4, a cDNA to human acidic ribosomal phosphoprotein PO mRNA (Laborda, 1991), was used to normalize RNA loading.

### In situ hybridization

Paraffin-embedded, 5-μm human breast tissue sections corresponding to normal tissue and tumour specimens were subjected to RNA in situ hybridization. A 258-bp fragment of human S100A2 cDNA obtained from the Huma 123 subtracted library (bases 103 to 360 inclusive of GenBank accession number M87068), cloned into pBluescript $KS^-$ was used as a template to generate Digoxigenin (DIG)-labelled sense and antisense cRNA by in vitro transcription from the flanking T7 and T3 RNA polymerase promoter sites in the pBluescript vector using a DIG-labelling system (Roche Molecular Biochemicals, Mannheim, Germany) according to the manufacturer's methods. The yields of DIG-labelled cRNA were estimated by spotting diluted aliquots of the labelled cRNA onto filters and analysing them with a DIG-labelled control RNA using a nitro-blue tetrazolium (NBT)/5-bromo-4-chloro-3-indolyl-phosphate (BCIP) colorimetric DIG-detection system (Roche Molecular Biochemicals, Mannheim, Germany). One μg of linearized plasmid DNA template normally generated about 10 μg of DIG-labelled cRNA probe which was stored as aliquots at −80˚C without observable degradation.

© 2000 Cancer Research Campaign

The tissue sections were de-waxed in fresh xylene, rehydrated with a graded series of ethanol solutions, washed once with 1 × phosphate-buffered saline (PBS) buffer (140 mM NaCl, 2.7 mM KCl, 10 mM $Na_2HPO_4$ and 1.8 mM $KH_2PO_4$, pH 7.4) and post-fixed with diethyl pyrocarbonate (DEPC)-treated PBS containing 4% (w/v) paraformaldehyde (MERCK, Dorset, UK) for 30 min at 4˚C to preserve the mRNA. The sections were treated with 200 mM HCl to denature the protein, and the tissue was acetylated in 0.5% (v/v) acetic anhydride (Sigma, St. Louis, USA) in 100 mM Tris-HCl pH 8.0 to reduce non-specific background. The sections were incubated with 20 μg $ml^{-1}$ RNase-free Proteinase K (Roche Molecular Biochemicals, Mannheim, Germany) in 50 mM Tris-HCl, pH 7.6, buffer containing 150 mM NaCl and 2 mM $CaCl_2$ for 30 min at 37˚C. After being washed with PBS, the sections were dehydrated in a series of ethanol solutions and the slides rinsed briefly with chloroform. After prehybridization in a solution of 5 × SSPE, 50% (v/v) deionized formamide and 5 × Denhardt's reagent at 42˚C for 1 h, each section was incubated with 200 μl or 500 μl (depending on the size of the section) of prehybridization buffer containing 5 mM dithiothreitol, 5% (w/v) dextran sulphate, 100 μg $ml^{-1}$ sheared, denatured salmon sperm DNA, and 200 ng $ml^{-1}$ of DIG-labelled antisense or sense cRNA for 16–18 h at 42˚C. After being washed at room temperature for 15 min with 2 × SSC (1 × SSC = 150 mM NaCl, 15 mM sodium citrate, pH 7.0), for 15 min with 1 × SSC, and for 30 min at 50˚C with 0.2 × SSC and 50% (v/v) deionized formamide, the slides were washed at room temperature for 15 min with 0.1 × SSC. Colour was developed using anti-Digoxigenin Fab fragments (from sheep), conjugated to alkaline phosphatase and the NBT/BCIP colorimeric detection system (Roche Molecular Biochemicals, Mannheim, Germany), according to the manufacturer's recommendations. The stained sections were examined by two independent observers, and the results were expressed as for immunocytochemistry above.

### Reverse transcription multiplex PCR analysis for S100A2 and glyceraldehyde 3-phosphate dehydrogenase cDNAs

Two μg of total RNA was reverse transcribed in a final volume of 10 μl with 200 units of SuperScript™ RNase $H^-$ Reverse Transcriptase (Life Technologies Ltd, Paisley, UK), and subsequently 1 μl of this first strand cDNA reaction mixture was amplified by PCR with *Taq* DNA Polymerase (Life Technologies Ltd, Paisley, UK). For S100A2 cDNA the forward primer (5′ position at base 103, of GenBank accession number M87068) was 5′-CCAAGAGGGCGACAAGTTCAAG-3′ and the reverse primer (5′ position at base 339) was 5′-CATGGCAGGGAGTCAAGAGTTC-3′. For the human glyceraldehyde 3-phosphate dehydrogenase cDNA the forward, 5′-ACCACAGTCCATGCCATCAC-3′ and reverse primer, 5′-TCCACCACCCTGTTGCTGTA-3′ were used to provide a normalization control. Multiplex PCR was performed with the following parameters: 94˚C for 5 min; 22 cycles at 94˚C for 30 s, 65˚C for 30 s and 72˚C for 2 min. PCR products were visualized with ethidium bromide following agarose gel electrophoresis (Sambrook et al, 1989).

### DNA sequencing

All DNA sequencing was performed using an automated ABI 377 DNA sequencing system and a standard dye terminator AmpliTaq kit (Perkin Elmer, Buckinghamshire, UK). The resulting sequences were analysed for homology in the public GenBank/EMBL/DDBJ/PDB and Expressed Sequence Tag (EST) databases using the basic local alignment search tool (BLAST) program (http://www.ncbi.nlm.nih.gov/BLAST) (Altschul et al, 1990).

### RESULTS

A subtracted library was constructed which contained cloned cDNAs representing mRNAs present at a higher level in a cell line (Huma 123) derived from benign breast disease than in the malignant breast tumour-derived cell line, MCF-7A and screened by nucleotide sequencing of 128 randomly-picked clones (approximately 10% of the library). Figure 1 shows the pattern of reverse Northern hybridization of a panel of just 12 of these cDNAs from the library including the cDNA for ribosomal protein L6, an abundant mRNA which is not differentially expressed to a large extent. The PCR-based suppression subtraction procedure yields some differentially expressed cloned cDNAs that are not easily detected in the reverse Northern screen (clones 3–7, 11, 12, Figure 1) and more-abundant cDNAs (clones 1, 2, 8 and 10) which are easily detected. Amongst these easily-detected cDNAs were those for plasminogen activator inhibitor I (11.6-fold elevated in the Huma 123 cells), annexin VIII (10.5-fold) and S100A2. This latter cDNA (number 1 in Figure 1), exhibited 100% identity to the sequence of human S100A2, a member of the S100 family of EF-hand proteins (Donato, 1999). Quantitation of the reverse Northern hybridization experiments using as probes single-stranded cDNAs from the Huma 123 or the MCF-7A cells confirmed that the level of S100A2 mRNA was at least 20-fold higher in the RNA from Huma 123 cells than in RNA from the MCF-7A cells (Figure 1), and that this degree of difference is greater than for some other differentially expressed mRNAs (Figure 1D), for example that of plasminogen activator inhibitor (11.6-fold).

S100A2 has been previously reported to be present in normal mammary epithelial tissue, but to be absent in breast cancer derived cells (Lee et al, 1992). Its abundance in a library from benign breast disease subtracted with RNA from the more malignant MCF-7A cells suggests that S100A2 mRNA might also be present in benign tumour-derived cells. A DNA probe corresponding to the S100A2 cDNA isolated from the subtracted library hybridized to a single highly abundant mRNA of 0.45 kb in Huma 123 RNA, confirming the expression of S100A2 mRNA in this cell line (Figure 2). Two additional faint hybridization bands at 4 kb and 2.5 kb in this lane (signified by arrows in Figure 2) probably correspond to unprocessed mRNA, one band containing both of the two introns (intron 1 of 1623 bp and intron 2 of 1961 bp) of the human S100A2 gene, and the other containing only intron 2 (Wicki et al, 1997). The Northern blotting experiments also show that the mRNA for S100A2 is present in a myoepithelial-like elongated convert, Huma 109, derived from the cell line Huma 123 and in an SV40-virus-immortalized cell line, Huma 7, derived from normal human mammary gland, but at a lower level relative to a constitutive mRNA than in the Huma 123 line (Figure 2). S100A2 mRNA is undetectable in RNA from malignant breast cancer cell lines, MCF-7A, T-47D, ZR-75 and MDA-MB-231 using standard Northern blotting procedures (Figure 2).

In order to examine the occurrence of S100A2 mRNA in tumour specimens, RT-PCR was used to amplify S100A2 mRNA specifically from RNA prepared from benign lesions and malignant human breast carcinoma specimens. Control amplifications of

© 2000 Cancer Research Campaign

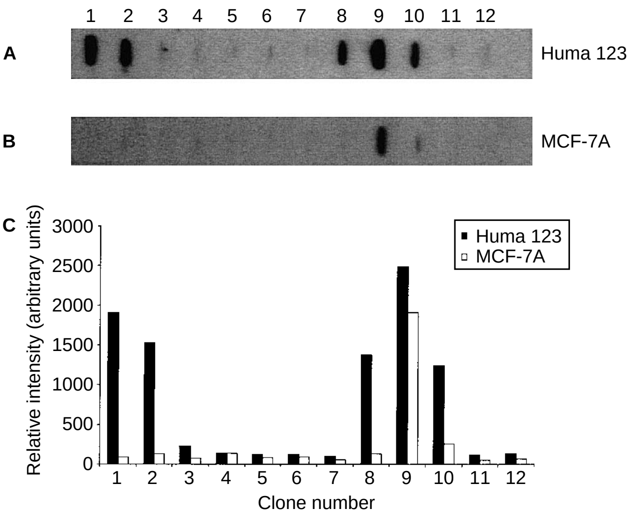

D

| Clone No. | Blast search | Accession No. |
|---|---|---|
| 1 | Human S100A2 mRNA | M87068 |
| 2 | Human PAI-1 mRNA | X04744 |
| 3 | Human mRNA for ets-2 | J04102 |
| 4 | Human SHC mRNA | X68148 |
| 5 | Human protocadherin Fat 2 mRNA | AF231022 |
| 6 | Human TNFR2-TRAF mRNA | L49432 |
| 7 | Human XIST mRNA | M97168 |
| 8 | Human anexin VIII mRNA | M81844 |
| 9 | Human mRNA for ribosomal protein L6 | M69391 |
| 10 | Human mRNA for p cadherin | M63629 |
| 11 | Unknown gene | AF257108 |
| 12 | Human MEN1 region mRNA | AF001893 |

**Figure 1** Reverse Northern hybridization of clones selected randomly from a cDNA library from a benign mammary derived cell line, Huma 123, suppression subtracted with driver cDNA derived from MCF-7A cells. 11 randomly-picked cloned cDNAs from the Huma 123 library were amplified by PCR, spotted onto duplicate Nylon filters and each filter hybridized separately with mixed cDNA probes of equal specific activity derived from Huma 123 RNA (Panel A) or MCF-7A RNA (Panel B), as described in Materials and Methods. The intensities of the autoradiographic images in (**A**) and (**B**) were quantified as described in Materials and Methods (Panel **C**). The cDNA for ribosomal protein L6 is included as a control for a cDNA which shows similar expression in the Huma 123 and MCF-7A cell lines. Panel **D** tabulates the identities of the cloned cDNAs 1–12 (Panels **A**–**C**) from their nucleotide sequence data. Clone 11 has been submitted to the GenBank database (Accession number AF257108). Human PAI-1 is Human plasminogen activator inhibitor 1 (Wun and Kretzmer, 1987); human *ets*-2 is human erythroblastosis virus oncogene homologue 2 mRNA (Watson et al, 1988); human SHC is the Src homology 2 (SH2) domain-containing transforming protein (Pelicci et al, 1992); human TNFR2-TRAF is the tumour necrosis factor receptor 2 (TNFR2)–TNF receptor associated factors (TRAF) signalling complex protein (Rothe et al, 1995); human XIST is human X (inactive)-specific transcript (Willard et al, 1992); human MEN1 is Multiple endocrine neoplasia type 1 protein (Guru et al, 1997)

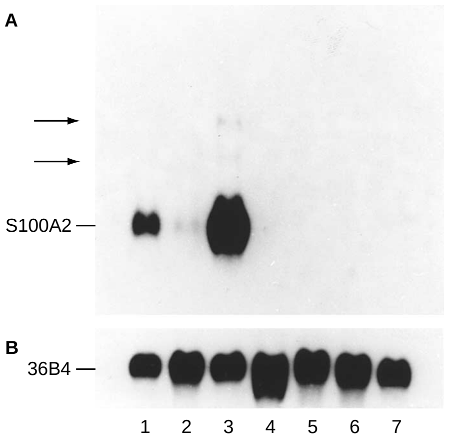

**Figure 2** Northern hybridization of S100A2 mRNA from human mammary cell lines derived from benign and malignant breast tumours. 10 μg of RNA from each of the SV40-immortalized human mammary cell line, Huma 7 (lane 1), the benign mammary derived cell line Huma 123 (lane 3), an elongated converted cell line, Huma 109 (lane 2), derived from Huma 123, the malignant mammary epithelial cell lines MCF-7A (lane 4), T-47D (lane 5), ZR-75 (lane 6) and MDA-MB-231 (lane 7) were subjected to Northern blotting and hybridization as described in Materials and Methods using $^{32}$P-labelled probes to S100A2 mRNA (Panel **A**) or to ribosomal phosphoprotein, 36B4 (Panel **B**) for normalization purposes. The arrows point to faint bands of hybridization in the Huma 123 RNA

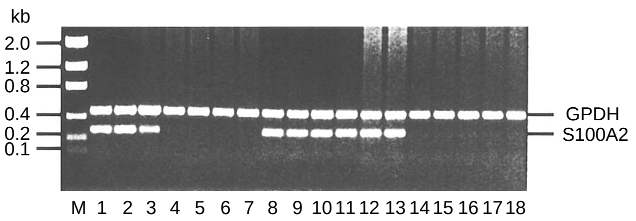

**Figure 3** The occurrence of S1002 mRNA in RNA from benign and malignant human mammary cell lines and breast tumour specimens by RT-PCR. RNA from normal and benign human mammary cell lines, Huma 7 (lane 1), Huma 123 (lane 2), Huma 109 (lane 3), and breast carcinoma-derived cell lines, MCF-7A (lane 4), T-47D (lane 5), ZR-75 (lane 6), MDA-MB-231 (lane 7), benign breast specimens (lanes 8–13) and breast carcinoma specimens (lanes 14–18) was amplified by RT-PCR using primers specific for S100A2 (S100A2) and human glyceraldehyde-3-phosphate dehydrogenase (GPDH) mRNA to yield PCR products of 258 bp and 452 bp, respectively. The resulting RT-PCR products were subjected to agarose gel electrophoresis and stained with ethidium bromide, as described in Materials and Methods. Lane M, DNA molecular weight markers cDNA derived from RNA from the cell lines (Figure 3) show that S100A2 mRNA was amplified from RNA from cell lines derived from normal and benign human mammary epithelium, but not from cell lines derived from breast carcinomas when 22 cycles of PCR were used. When PCR was carried out using this same number of cycles, amplification of S100A2 mRNA was evident in RNA from specimens of benign disease, but not from specimens of breast carcinomas (Figure 3).

In order to identify whether the S100A2 mRNA arose from normal or abnormal epithelium within the benign disease specimens, histological sections of benign specimens were subjected to in situ hybridization, using sense and antisense RNA probes specific for S100A2 mRNA. Antisense probes hybridized strongly to the ductal and lobular epithelial components, but not to the stromal components on sections of normal human breast, whereas sense probes failed to hybridize, even though tissue was present in the same field (Figure 4). The absence of a signal with the sense probe, and the fact that the antisense probe failed to stain the nuclei of the cells indicates that there was no hybridization of the probe to genomic DNA (Figure 4).

When histological sections from a panel of 27 carcinoma specimens were examined by in situ hybridization, there was no staining of the majority (23/27) of the specimens (Figure 4; Table 2), however, there was weak staining in 4 carcinoma specimens (Table 2). Of the 4 carcinoma specimens which contained low levels of S100A2 mRNA, 2 were estrogen receptor positive and

 *© 2000 Cancer Research Campaign*

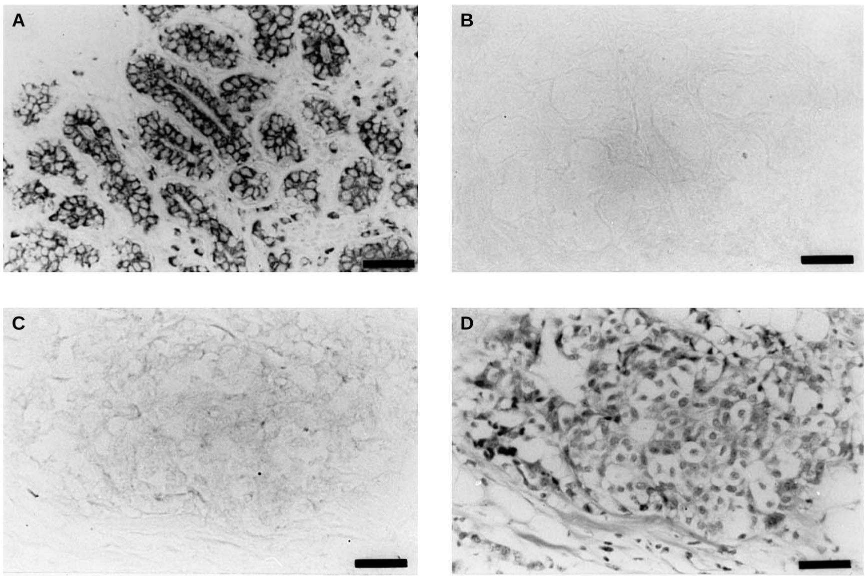

**Figure 4** In situ hybridization of S100A2 mRNA in normal human mammary gland and in a human breast carcinoma. Histological sections of normal mammary gland (Panels **A** and **B**) and a carcinoma specimen (panel **C**) were subjected to in situ hybridization using anti-sense (Panels **A** and **C**) or sense (Panel **B**) probes to S100A2 mRNA as described in Materials and Methods. An adjacent histological section from the carcinoma specimen was stained with haematoxylin and eosin (Panel **D**). Magnification is ×206; bars = 50 μm.

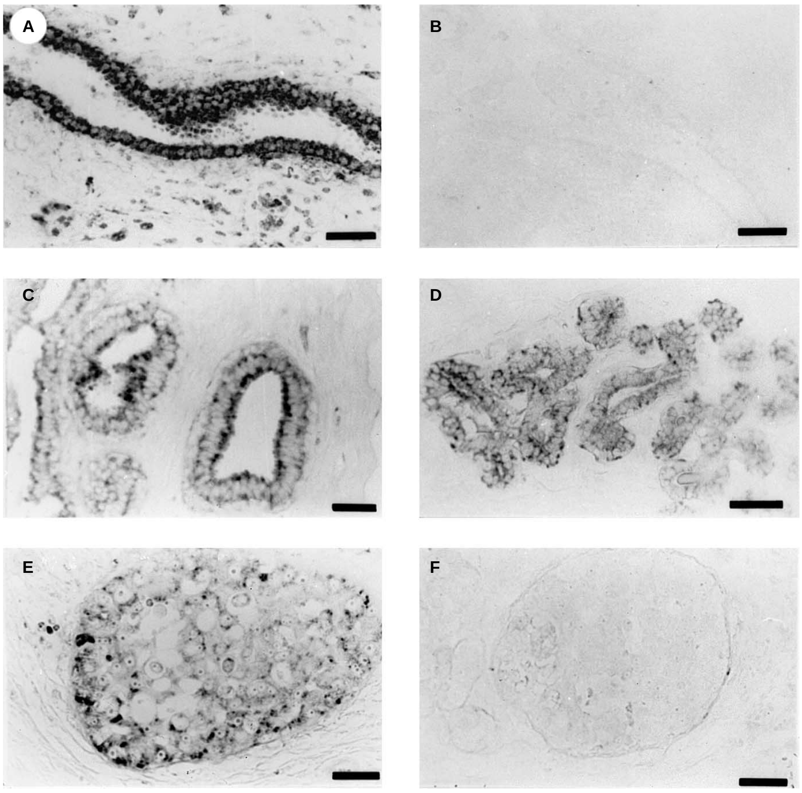

**Figure 5** In situ hybridization of S100A2 mRNA in benign human breast lesions and in ductal carcinomas in situ. Histological sections of benign tumours, of fibroadenoma (Panels **A**, **B**), of hyperplasia (Panels **C**, **D**), or of an S100A2-positive (Panel **E**) and an S100A2-negative (Panel **F**) ductal carcinoma in situ were subjected to in situ hybridization with anti-sense (Panels **A** and **C**–**F**) or sense (Panel **B**) probes as described in Materials and Methods. Magnification is ×206 (Panels **A**–**D**) or ×165 (Panels **E** and **F**). Bars = 50 μm (Panels **A**–**D**) or 60 μm (Panels **E** and **F**).

node negative, one was estrogen receptor positive and of unknown nodal status, and one was estrogen receptor positive node positive.

When benign breast tumour and hyperplastic specimens were subjected to in situ hybridization, the epithelial cells surrounding the abnormal ductal structures stained strongly for S100A2 mRNA in all 15 specimens (Figure 5, Table 2), although a comparison of the staining of normal and benign structures on the same sections suggested that in some sections the benign specimens stained slightly less strongly than the normal ductal structures (not shown).

© 2000 Cancer Research Campaign

**Table 2** Staining of tumour specimens for S100A2 mRNA by in situ hybridization

| Tissue specimen | In situ hybridization for S100A2 mRNA | | |
|---|---|---|---|
| | Total number of specimens | Number of S100A2 positive specimens | Number of S100A2 negative specimens |
| Normal gland | 3 | 3 | 0 |
| Normal region of tumour specimens | 5 | 5 | 0 |
| Benign lesions | 15 | 15 | 0 |
| Carcinoma in situ[a] | 19 | 7 | 12 |
| p53 positive | 8 | 2 | 6 |
| p53 negative[b] | 9 | 4 | 5 |
| Unknown p53 | 2 | 1 | 1 |
| Carcinoma[c] | 27 | 4 | 23 |
| p53 positive | 12 | 0 | 12 |
| p53 negative[d] | 11 | 3 | 8 |
| Unknown p53 | 4 | 1 | 3 |

[a]Significantly different from normal ($P$ = 0.0096) or benign ($P$ = 0.005) specimens (Fisher Exact Test). Not significantly different from carcinoma specimens ($P$ >0.16; Fisher Exact Test). [b]Not significantly different from p53-positive carcinoma in situ specimens ($P$ = 0.742; Fisher Exact Test). [c]Significantly different from normal ($P$ < 0.0001) or benign ($P$ = 0.0001) specimens (Fisher Exact Test). Not significantly different from carcinoma in situ specimens ($P$ > 0.16; Fisher Exact Test). [d]Not significantly different from p53-positive carcinoma in situ specimens ($P$ = 0.187; Fisher Exact Test).

In contrast to the benign tumour and hyperplastic specimens, the staining of carcinoma in situ was much more variable (Figure 5, Table 2). Only 7/19 (37%) of the specimens of carcinoma in situ examined stained positively for S100A2 mRNA, whilst the remaining 12 specimens exhibited a complete absence of S100A2 mRNA (Figure 5; Table 2). Those specimens which stained positively showed a patchy staining which was generally less intense than the staining of either normal or benign epithelial cells (Figure 5E).

### DISCUSSION

Amongst cDNAs expressed at a markedly higher level in the benign human mammary cell line, Huma 123, than in the malignant MCF-7 cells were annexin VIII, plasminogen activator inhibitor-1 and S100A2. This is the first report of down-regulation of annexin VIII in human malignant breast cells, however, annexin VIII has previously been identified as being up-regulated in acute promyelocytic leukaemias (APL) but not in other leukaemic cells. This up-regulation is thought to be a result of the down-regulation of the retinoic acid receptor in promyelocytic leukaemia (Chang et al, 1992). Thus, in the mammary cells, the differential expression of annexin VIII might also reflect changes in the function of the retinoic acid receptor.

S100A2 mRNA has been found to be expressed abundantly in benign human breast cell lines and, as reported previously (Lee et al, 1992), to be undetectable by Northern blotting in cell lines derived from breast carcinoma cell lines, MCF-7A, ZR-75, T-47D. The cell line MDA-MB-231 was similarly negative, however, this latter cell line has been previously shown to contain S100A2 mRNA (Pedrocchi et al, 1994) and protein, which has been reported to be located in the nucleus in these cells (Mueller et al, 1999). In the present experiments, a faint band of hybridization was detectable in RNA from the MDA-MB-231 cells when Northern blots were highly overexposed suggesting the presence of low levels of S100A2 mRNA in these particular cells.

Using in situ hybridization, S100A2 mRNA has been shown to be present in normal human breast epithelium and to be generally absent in breast carcinoma specimens, as expected. However, unexpectedly, there was a very low level of staining for S100A2 mRNA in a small proportion (4/27) of breast carcinoma specimens, a result that is entirely consistent with the detection of low levels of S100A2 mRNA in one of 4 malignant breast carcinoma cell lines.

It has previously been suggested that S100A2 is up-regulated upon stimulation of p53-dependent apoptosis (Wang et al, 1999), and in transactivation assays it has been shown that the S100A2 promoter can be transcriptionally activated by wild-type, but not by mutant, p53 (Tan et al, 1999). In the present group of specimens, it seems unlikely that the down-regulation of S100A2 arises from the presence of mutant, and therefore inactive, p53 as indicated by the presence of nuclear staining for p53, since 8 S100A2-negative specimens were also negative for p53, suggesting a normal p53 phenotype in these specimens. Apart from the possibility that these carcinoma specimens exhibited a p53 null phenotype due to deletion of both copies of the p53 gene, it is likely that the loss of S100A2 in the majority of carcinoma cells is due to other mechanisms, such as methylation of the S100A2 gene (Lee et al, 1992; Wicki et al, 1997).

The present experiments are the first report of S100A2 mRNA occurring in benign breast specimens both in benign tumours and in hyperplastic lesions. Not only was S100A2 mRNA present in 100% of 15 benign tumour and hyperplastic lesions examined, but also the staining was hardly less than that in the normal mammary parenchyma. This is the first report of the presence of S100A2 in benign breast neoplastic or hyperplastic cells, although S100A2 mRNA has been shown previously to occur in benign naevi of the skin (Maelandsmo et al, 1997). Since S100A2 mRNA is almost completely absent in the breast carcinoma cells examined, the results of the present experiments suggest that S100A2 mRNA becomes down-regulated during the formation of carcinoma cells, at least in the breast, and not at the earlier stage of the development of benign hyperplastic and neoplastic cells from normal epithelium. These results suggest that S100A2 is not a simple suppressor of tumour cell growth (Lee et al, 1992), but it might be the result of tumour progression or otherwise associated with it.

Furthermore, S100A2 mRNA is present in the carcinoma cells in only 37% of carcinoma in situ examined and that, in these

 *© 2000 Cancer Research Campaign*

specimens, the proportion of cells staining is considerably reduced relative to the benign lesions. Since S100A2, uniquely amongst the S100A proteins, has been suggested to have a nuclear localization in some cells, for example, human vascular smooth muscle cells (Mandinova et al, 1998), and in the Huma 7 breast epithelial cells (Elenis and Wang, personal communication), one of the cell lines used in the present study, it is possible that the loss of S100A2 is associated with changes in the nucleus which reflect the progression to the malignant phenotype in human breast carcinomas.

### ACKNOWLEDGEMENTS

We thank Clatterbridge Cancer Research Trust for a studentship to Dong Liu and for supporting this research. Additional financial support from the Cancer and Polio Research Fund, and an ORS award to Dong Liu from HEFCE are gratefully acknowledged. We thank the CANDIS Cancer Tissue Bank Research Centre for providing some tissue specimens and Ioannis Elenis and Guozheng Wang for access to unpublished results.

### REFERENCES

Altschul S, Gish W, Miller W, Myers E and Lipman D (1990) Basic local alignment search tool. *J Mol Biol* **215**: 403–410

Ambartsumian N, Grigorian M, Larsen F, Karlstrom O, Sidenius N, Rygaard J, Georgiev G and Lukanidin E (1996) Metastasis of mammary carcinomas in GRS/A hybrid mice transgenic for the *mts*1 gene. *Oncogene* **13**: 1621–1630

Anandappa S, Winstanley J, Leinster S, Green B, Rudland P and Barraclough R (1994) Comparative expression of fibroblast growth factor mRNAs in benign and malignant breast disease. *Br J Cancer* **69**: 772–776

Barraclough R, Kimbell R and Rudland PS (1987) Differential control of mRNA levels for Thy-1 antigen and laminin in rat mammary epithelial and myoepithelial-like cells in culture. *J Cell Physil* **131**: 393–401

Briand P, Petersen OW and Van Deurs B (1987) A new diploid nontumorigenic human breast epithelial cell line isolated and propagated in chemically-defined medium. *In vitro* **23**: 181–188

Chang K, Wang G, Freireich E, Daly M, Naylor S, Trujillo J and Stass S (1992) Specific expression of the annexin VIII gene in acute promyeloytic leukaemia. *Blood* **79**: 1802–1810

Chirgwin JM, Przybyla AE, Macdonald RJ and Rutter WJ (1979) Isolation of biologically active ribonucleic acid from sources enriched in ribonuclease. *Biochemistry* **18**: 5294–5299

Davies B, Davies M, Gibbs F, Barraclough R and Rudland P (1993) Induction of the metastatic phenotype by transfection of a benign rat mammary epithelial cell line with the gene for p9Ka, a rat calcium-binding protein but not with the oncogene EJ *ras*-1. *Oncogene* **8**: 999–1008

Davies M, Rudland P, Robertson L, Parry E, Jolicoeur P and Barraclough R (1996) Expression of the calcium-binding protein S100A4 (p9Ka) in MMTV-*neu* transgenic mice induces metastasis of mammary tumours. *Oncogene* **13**: 1631–1637

Diatchenko L, Lau Y-FC, Campbell A, Chenchik A, Moqadaam F, Huang B, Lukyanov S, Lukyanov K, Gurskaya N, Sverdlov E and Siebert P (1996) Suppression subtractive hybridisation: a method for generating differentially-regulated or tissue-specific cDNA probes and libraries. *Proc Natl Acad Sci USA* **93**: 6025–6030

Donato R (1999) Functional roles of S100 proteins, calcium-binding proteins of the EF-hand type. *Biochim Biophys Acta* **1450**: 191–231

Engel LW and Young NA (1978) Human breast carcinoma cells in continuous culture: a review. *Cancer Res* **38**: 4327–4339

Feinberg AP and Vogelstein B (1984) A technique for radiolabelling DNA restriction endonuclease fragments to high specific activity. *Anal Biochem* **137**: 266–267

Guru SC, Agarwal SK, Manickam P, Olufemi S-E, Crabtree JS, Weisemann JM, Kester M, Kim YS, Emmert-Buck MR, Liotta LA, AM, S, Boguski M, Roe BA, Collins FS, Burns AL, Marx SJ and Chandrasekharappa SC (1997) A transcript map for the 2.8-Mb region containing the multiple endocrine neoplasia type 1 locus. *Genome Res* **7**: 725–735

Han JH, Stratowa C and Rutter WJ (1987) Isolation of full-length putative rat lysophospholipase cDNA using improved methods for mRNA isolation and cDNA cloning. *Biochemistry* **26**: 1617–1625

Ke Y, Fernig DG, Wilkinson MC, Winstanley JHR, Smith JA, Rudland PS and Barraclough R (1993) The expression of basic fibroblast growth factor and its receptor in cell lines derived from normal human mammary gland and a benign mammary lesion. *J Cell Sci* **106**: 135–143

Laborda J (1991) 36B4 cDNA used as an estradiol-independent mRNA control is the cDNA for human acidic ribosomal phosphoprotein PO. *Nucleic Acids Res* **19**: 3998

Lee SW, Tomasetto C, Swisshelm K, Keyomarsi K and Sager R (1992) Down-regulation of a member of the S100 gene family in mammary-carcinoma cells and reexpression by azadeoxycytidine treatment. *Proc Natl Acad Sci USA* **89**: 2504–2508

Maelandsmo GM, Florenes VA, Mellingsaeter T, Hovig E, Kerbel RS and Fodstad O (1997) Differential expression patterns of S100A2, S100A4 and S100A6 during progression of human malignant melanoma. *Int J Cancer* **74**: 464–469

Mandinova A, Atar D, Schafer BW, Spiess M, Aebi U and Heizmann CW (1998) Distinct subcellular localization of calcium binding S100 proteins in human smooth muscle cells and their relocation in response to rises in intracellular calcium. *J Cell Sci* **111**: 2043–2054

Mueller A, Bachi T, Hochli M, Schäfer BW and Heizmann CW (1999) Subcellular distribution of S100 proteins in tumor cells and their relocation in response to calcium activation. *Histochem Cell Biol* **111**: 453–459

Pedrocchi M, Schäfer BW, Mueller H, Eppenberger U and Heizmann CW (1994) Expression of $Ca^{2+}$-binding proteins of the S100 family in malignant human breast-cancer cell-lines and biopsy samples. *Int J Cancer* **57**: 684–690

Pelicci G, Lanfrancone L, Grignani F, McGlade J, Cavallo F, Forni G, Nicoletti I, Grignani F, Pawson T and Pelicci PG (1992) A novel transforming protein (SHC) with an SH2 domain is implicated in mitogenic signal transduction. *Cell* **70**: 93–104

Platt-Higgins A, Renshaw C, West C, Winstanley J, De Silva Rudland S, Barraclough R and Rudland P (2000) Comparison of the metastasis-inducing protein S100A4 (p9Ka) with other prognostic markers in human breast cancer. *Int J Cancer* **89**: 198–208

Rothe M, Pan MG, Henzel WJ, Ayres TM and Goeddel DV (1995) The TNFR2-TRAF signaling complex contains two novel proteins related to baculoviral inhibitor of apoptosis proteins. *Cell* **83**: 1243–1252

Rudland PS, Ollerhead G and Barraclough R (1989) Isolation of simian virus 40 transformed human mammary epithelial stem cell line that can differentiate to myoepithelial-like cells in culture and *in vivo. Develop Biol* **136**: 167–180

Rudland P, Platt-Higgins A, Renshaw C, West C, Winstanley J, Robertson L and Barraclough R (2000) Prognostic significance of the metastasis-inducing protein S100A4 (p9Ka) in human breast cancer. *Cancer Res* **60**: 1595–1603

Sambrook J, Fritsch EF and Maniatis T (1989) *Molecular Cloning: A Laboratory Manual.* Cold Spring Harbor Laboratory Press: Cold Spring Harbor, NY

Schäfer B and Heizmann C (1996) The S100 family of EF-hand calcium-binding proteins: function and pathology. *TIBS* **21**: 134–140

Tan MJ, Heizmann CW, Guan KL, Schäfer BW and Sun Y (1999) Transcriptional activation of the human S100A2 promoter by wild-type p53. *FEBS Lett* **445**: 265–268

Taylor S, Platt-Higgins A, Rudland P, Winstanley J and Barraclough R (1997) Cytoplasmic staining of c-*erb*B-2 is not associated with the presence of detectable c-*erb*B-2 mRNA in breast cancer specimens. *Int J Cancer* **76**: 459–463

Wang YX, Rea T, Bian JH, Gray S and Sun Y (1999) Identification of the genes responsive to etoposide-induced apoptosis: application of DNA chip technology. *FEBS Lett* **445**: 269–273

Watson DK, McWilliams MJ, Lapis P, Lautenberger JA, Schweinfest CW and Papas TS (1988) Mammalian *ets*-1 and *ets*-2 genes encode highly conserved proteins. *Proc Natl Acad Sci U.S.A.* **85**: 7862–7866

Wicki R, Franz C, Scholl FA, Heizmann CW and Schäfer BW (1997) Repression of the candidate tumor suppressor gene S100A2 in breast cancer is mediated by site specific hypermethylation. *Cell Calcium* **22**: 243–254

Willard HF, Lawrence J, Xing Y, Lafreniere RG, Rupert JL, Hendrich BD and Brown CJ (1992) The human XIST gene: analysis of a 17 kb inactive X-specific RNA that contains conserved repeats and is highly localized within the nucleus. *Cell* **71**: 515–529

Wun TC and Kretzmer KK (1987) cDNA cloning and expression in *E. coli* of a plasminogen activator inhibitor (PAI) related to a PAI produced by Hep G2 hepatoma cell. *FEBS Lett* **210**: 11–16

© 2000 Cancer Research Campaign
